# Hesitant Fuzzy Linguistic Multicriteria Decision-Making Method Based on Generalized Prioritized Aggregation Operator

**DOI:** 10.1155/2014/645341

**Published:** 2014-09-01

**Authors:** Jia-ting Wu, Jian-qiang Wang, Jing Wang, Hong-yu Zhang, Xiao-hong Chen

**Affiliations:** School of Business, Central South University, Changsha 410083, China

## Abstract

Based on linguistic term sets and hesitant fuzzy sets, the concept of hesitant fuzzy linguistic sets was introduced. The focus of this paper is the multicriteria decision-making (MCDM) problems in which the criteria are in different priority levels and the criteria values take the form of hesitant fuzzy linguistic numbers (HFLNs). A new approach to solving these problems is proposed, which is based on the generalized prioritized aggregation operator of HFLNs. Firstly, the new operations and comparison method for HFLNs are provided and some linguistic scale functions are applied. Subsequently, two prioritized aggregation operators and a generalized prioritized aggregation operator of HFLNs are developed and applied to MCDM problems. Finally, an illustrative example is given to illustrate the effectiveness and feasibility of the proposed method, which are then compared to the existing approach.

## 1. Introduction

Since the fuzzy set was proposed by Zadeh in 1965 [1], it has been widely researched and developed as well as being successfully applied in various fields [2–5]. Due to the fuzziness and uncertainty of many MCDM problems, the criteria weights and values of alternatives can be inaccurate, uncertain, or incomplete. Under such circumstances, Zadeh's fuzzy sets can provide robust solutions. In Zadeh's fuzzy sets, the membership degree of the element in a universe is a single value between zero and one; however, these single values are inadequate to provide complete information due to a lack of systematic and comprehensive knowledge.

Hesitant fuzzy sets (HFSs), an extension of traditional fuzzy sets, can address this problem. HFSs were first introduced by Torra and Narukawa [6, 7], and they permit the membership degree of an element to be a set of several possible values between zero and one. HFSs are highly useful in expressing hesitance existing when decision makers give the evaluation values, and they have been a subject of great interest to researchers. For example, some work on the aggregation operators of HFSs has been undertaken in [8–11], and the distance and correlation measures for HFSs were developed in [12–15]. Later still, hesitant fuzzy TOPSIS [16] and hesitant fuzzy TODIM [17] methods for solving MCDM problems have been proposed.

When faced with problems that are too complex or ill-defined to be solved by quantitative expressions, linguistic variables can be an effective tool because the use of linguistic information enhances the reliability and flexibility of classical decision models [18]. Linguistic variables have been studied in depth and used in many fields [19–23]. The linguistic variable could be a single linguistic term [24], or interval of linguistic terms, that is, uncertain linguistic variables [25]. Rodríguez et al. [26, 27] proposed hesitant fuzzy linguistic term sets (HFLTSs) that assess a linguistic variable by using several linguistic terms. However, similar to linguistic variables, they cannot reflect the possible membership degrees of a linguistic term to a given concept. The information they express is not sufficiently comprehensive and they cannot deal with problems in which both the evaluation value and its associate membership degrees are described through fuzzy concepts. By contrast, intuitionistic linguistic sets (ILSs) [28] and their extensions [29, 30] can describe two fuzzy attributes of an object: a linguistic variable and an intuitionistic fuzzy number. The former provides an evaluation value, whilst the latter describes the confidence degree for the given evaluation value.

To express decision-makers' hesitance that exists in giving the associated membership degrees of one linguistic term, the concept of hesitant fuzzy linguistic sets (HFLSs), which is based on linguistic term sets and HFSs, was introduced in [31]. The elements in HFLSs are called hesitant fuzzy linguistic numbers (HFLNs). That is to say, for one object, an HFLS is reduced to an HFLN, which can be considered as a special case of HFLSs. For example, 〈*s*_2_, (0.3,0.4,0.5)〉 is an HFLN and 0.3, 0.4, and 0.5 are the possible membership degrees to the linguistic term *s*_2_. HFLSs have enabled great progress in describing linguistic information and to some extent may be considered an innovative construct. The main advantage of HFLSs is that they can describe two fuzzy attributes of an object: a linguistic term and a hesitant fuzzy element (HFE). The former provides an evaluation value, such as “excellent” or “good.” The latter describes the hesitancy for the given evaluation value and denotes the membership degrees associated with the specific linguistic term. However, the operations proposed by Lin et al. [31] have some limitations that will be discussed in Section 3, and this paper will define new operations for HFLNs.

To date, several methods have been proposed for dealing with linguistic information and the main ones will now be briefly described. (1) One of the methods is based on a transformation to fuzzy numbers, which converts linguistic information into triangular, or other kinds of fuzzy numbers by means of a membership function [32–34]. However, this method led to a certain degree of information loss in the transformation process and it is difficult to choose the appropriate membership functions in practical decision-making applications. (2) Another method is based on symbols that made computations on the subscripts of linguistic terms and was easy to operate [35–38]. However, in order to express the results in the initial term sets, this method performed the retranslation step as an approximation process, which led to a lack of accuracy [39]. (3) One method is based on the cloud model, which can correctly depict the uncertainty of a qualitative concept. This model has been successfully utilized [40–42]. (4) Another method is based on the 2-tuple linguistic representation model [43], which avoided the information distortion and loss that had hitherto occurred in linguistic information processing [44–47]. In this method, there is a conversion and inverse conversion process. Motivated by this idea and taking into consideration the limitations in previous linguistic methods, Wang et al. [48] proposed linguistic scale functions to deal with linguistic translation issues under different semantic situations. These scale functions provide a higher degree of flexibility for modeling linguistic information.

In general, aggregation operators are important tools for dealing with information fusion in MCDM problems and are a research area of great interest throughout the world. In practical situations, decision makers usually consider different criteria priorities. To deal with this issue, Yager [49] developed prioritized average (PA) operators by modeling the criteria priority on the weights associated with criteria, which are dependent on the satisfaction of higher priority criteria. Yager [50] further focused on PA operators and proposed two methods for formulating this type of aggregation process. As is well known, the PA operator has many advantages over other operators. For example, the PA operator does not need to provide weight vectors and, when using this operator, it is only necessary to know the priority among criteria. However, Yager [49] only discussed the criteria values and weights in real number domain, and there has been no aggregation operator that considers different criteria priorities in the aggregation process for HFLNs. Therefore, the aim of this paper is to develop some PA operators for aggregating hesitant fuzzy linguistic information.

The paper will focus on a type of MCDM problems where criteria priority exists, referred to as a prioritized MCDM problem. Two PA operators and one generalized PA operator for HFLSs will be proposed under a hesitant fuzzy linguistic environment. These operators are mainly used for solving hesitant fuzzy linguistic MCDM problems in which the criteria are in different priority levels. Therefore, the rest of this paper is organized as follows. In Section 2, some basic concepts of linguistic term sets and HFSs are briefly reviewed. In Section 3, new operations of HFLNs are provided and a method for comparing two HFLNs is proposed based on the linguistic scale functions. In Section 4, the PA operators for HFLNs are proposed and some desirable properties are analyzed. Then, a method for solving MCDM problems with HFLNs, in which the criteria are in different priority levels, is developed. In Section 5, an illustrative example is provided and subsequently the comparison analysis is made. Finally, the conclusions are drawn in Section 6.

## 2. Preliminaries

Before discussing HFLSs, some related concepts, such as linguistic term sets and HFSs are reviewed in this section. These concepts can lead to a better understanding of HFLSs.

### 2.1. The Linguistic Term Sets and Their Extension

Let *S* = {*s*_*i*_∣*i* = 0,1,…, 2*t*} be a finite and linguistic term set with odd cardinality, where *s*_*i*_ represents a possible value for a linguistic variable and should satisfy the following characteristics [32].

1. The set is ordered: *s*_*i*_ > *s*_*j*_, if *i* > *j*.
2. There is a negation operator: *s*_*i*_ = neg(*s*_*j*_) satisfying *i* + *j* = 2*t*.

For example, when *t* = 3, a linguistic term set *S* could be given as follows:

(1) S={s0,s1,s2,s3,s4,s5,s6}={very  poor,poor,slightly  poor,fair,slightly  good,good,very  good}.

When aggregating information as part of the decision-making process, the aggregated results do not regularly match the elements in the language assessment scale. To preserve all the information provided, Xu [51, 52] extended the discrete linguistic term set *S* to a continuous linguistic term set $\overset{-}{S}=\{s_{i}|i\in [0,l]\}$, in which *s*_*i*_ > *s*_*j*_ if *i* > *j*, and *l*  (*l* > 2*t*) is a sufficiently large positive integer. If *s*_*i*_ ∈ *S*, then *s*_*i*_ is called the original linguistic term; otherwise, *s*_*i*_ is called the virtual linguistic term.

In general, decision makers use original linguistic terms to evaluate alternatives, whereas virtual linguistic terms are only used as part of the calculation process in order to avoid information loss and generally enhance the overall decision making [51]. Virtual linguistic terms have no practical meaning, with their main role being to rank the alternatives [53].

### 2.2. HFSs

**Definition 1 (see [6]).**

Let *X* be a reference set, and let a hesitant fuzzy set (HFS) on *X* be in terms of a function that will return a subset of [0,1] in the case of it being applied to *X*.

To be easily understood, Xia and Xu [54] expressed HFSs by a mathematical symbol:

(2) $$\begin{array}{c} E=\left\{\langle x,h_{E}\left(x\right)\rangle \;|\;x\in X\right\}, \end{array}$$

where *h*_*E*_(*x*) is a set of values in [0,1], denoting the possible membership degrees of the element *x* ∈ *X* to the set *E*. *h*_*E*_(*x*) is called a hesitant fuzzy element (HFE) [54].

**Example 2 .**

Let *X* = {*x*_1_, *x*_2_} be a universal set, and two HFEs *h*_*E*_(*x*_1_) = {0.1,0.4} and *h*_*E*_(*x*_2_) = {0.1,0.5,0.7}, respectively, denote the membership degrees of *x*_*i*_  (*i* = 1,2) to the set *E*. *E* is an HFS, where *E* = {〈*x*_1_, {0.1,0.4}〉, 〈*x*_2_, {0.1,0.5,0.7}〉}.

**Definition 3 (see [54]).**

For an HFE *h*, let *l*(*h*) be the number of values in *h*, and then *s*(*h*) = (1/*l*(*h*))∑_*γ*∈*h*_ *γ* is called the score function of *h*. For two HFEs *h*_1_ and *h*_2_, if *s*(*h*_1_) > *s*(*h*_2_), then *h*_1_ is superior to *h*_2_, denoted by *h*_1_≻*h*_2_; if *s*(*h*_1_) = *s*(*h*_2_), then *h*_1_ is indifferent to *h*_2_, denoted by *h*_1_ ~ *h*_2_.

## 3. HFLNs and Their Operations

HFLNs, as the elements and special case of HFLSs, have great significance for information evaluation. In this section, the advantages and applications of HFLNs are firstly introduced. Then, new operations and comparison laws of HFLNs, which will be used in the latter analysis, are also presented.

### 3.1. HFLSs

**Definition 4 (see [31]).**

Let *X* = {*x*_1_, *x*_2_,…, *x*_*n*_} be a fixed set and *s*_*θ*(*x*)_ ∈ *S*. An HFLS *A* in *X* is an object:

(3) $$\begin{array}{c} A=\left\{\langle x,s_{\theta (x)},h_{A}\left(x\right)\rangle \;|\;x\in X\right\}, \end{array}$$

where *h*_*A*_(*x*) is a set of finite numbers in [0,1] and denotes the possible membership degrees that *x* belongs to *s*_*θ*(*x*)_.

When *X* has only one element, the HFLS *A* is reduced to 〈*s*_*θ*(*x*)_, *h*_*A*_(*x*)〉. For computational convenience, we call *α* = 〈*s*_*θ*(*α*)_, *h*_*α*_〉 as an HFLN.

When *h*_*A*_(*x*) = {*r*} has only one element, it indicates that the degree that *x* belongs to *s*_*θ*(*x*)_ is *r*. For example, 〈*s*_2_, 0.3〉 is called a fuzzy linguistic number, which is a special case of HFLN.

**Example 5 .**

Let *X* = {*x*_1_, *x*_2_} be a universal set. If an HFLS *A* = {〈*x*, *s*_*θ*(*x*)_, *h*_*A*_(*x*)〉∣*x* ∈ *X*} = {〈*x*_1_, *s*_5_, {0.4,0.6,0.7}〉, 〈*x*_2_, *s*_6_, {0.1,0.5,0.7}〉} is divided into two subsets that contain only one object, respectively, then 〈*s*_5_, {0.4,0.6,0.7}〉 and 〈*s*_6_, {0.1,0.5,0.7}〉 are HFLNs. 0.4, 0.6, and 0.7 are the possible membership degrees that *x*_1_ belongs to *s*_5_; 0.1, 0.5, and 0.7 are the possible membership degrees that *x*_2_ belongs to *s*_6_.

An HFLN is an extension of a linguistic term and an HFE. Compared to linguistic terms, HFLNs embody the possible membership degrees that an evaluation object attaches to the linguistic term, and they can depict the fuzziness more accurately than an uncertain linguistic variable does. When compared to HFEs, HFLNs add linguistic terms and assign the membership function to a specific linguistic evaluation value, which make the membership degrees no longer relative to a fuzzy concept, but to linguistic terms, such as “poor” or “good.”

In fuzzy set theory, the hesitant values in HFLNs are called possible membership degrees, which are caused by the hesitancy and uncertainty of decision makers. In the example of the performance evaluation of a car, suppose that “good (*s*_5_)” is an acceptable evaluation result for the car and is given by three decision makers. Then, each decision maker uses a value to express his/her opinion about the car under the evaluation of “good (*s*_5_).” Decision maker A may give the value 0.4 for “good,” whilst decision maker B may give 0.6 and decision maker C may give 0.7. In this case, HFLNs may be a better choice, and the evaluation result can be denoted by 〈*s*_5_, {0.4,0.6,0.7}〉.

HFLSs and linguistic hesitant fuzzy sets (LHFSs) [55] are different concepts. An HFLS is defined on a finite set (a set of objects) *X* = {*x*_1_, *x*_2_,…, *x*_*n*_}, while an LHFS is defined for one object *y*. For example, *A* = {〈*x*_1_, *s*_2_, {0.6,0.8}〉, 〈*x*_2_, *s*_5_, {0.4,0.6}〉, 〈*x*_3_, *s*_6_, {0.1,0.5,0.7}〉} is an HFLS, and *B* = {〈*s*_2_, {0.6,0.8}〉, 〈*s*_5_, {0.4,0.6}〉, 〈*s*_6_, {0.1,0.5,0.7}〉} is an LHFS. In the HFLS *A*, 〈*s*_2_, {0.6,0.8}〉, 〈*s*_5_, {0.4,0.6}〉 and 〈*s*_6_, {0.1,0.5,0.7}〉 are evaluation values for objects *x*_1_, *x*_2_, and *x*_3_, respectively. In the LHFS *B*, 〈*s*_2_, {0.6,0.8}〉, 〈*s*_5_, {0.4,0.6}〉, and 〈*s*_6_, {0.1,0.5,0.7}〉 are considered as a whole, which are the evaluation values for *y*. To some extent, an LHFS can be considered to be composed of several HFLNs. The elements in *B*, that is, 〈*s*_2_, {0.6,0.8}〉, 〈*s*_5_, {0.4,0.6}〉, and 〈*s*_6_, {0.1,0.5,0.7}〉, are regarded as three HFLNs. Such a processing may distort the initial definitions of HFLNs and LHFSs but can make some useful operations and algorithm of HFLNs be feasible for LHFSs. In summary, LHFSs are more complex for experts or decision makers to express their preference than HFLNs because both the linguistic terms and their membership degrees in LHFSs are uncertain and inconsistent simultaneously.

### 3.2. Linguistic Scale Functions

To use data more efficiently and to express the semantics more flexibly, linguistic scale functions assign different semantic values to linguistic terms under different situations [48]. They are preferable in practice because these functions are flexible and can give more deterministic results according to different semantics.

**Definition 6 (see [48]).**

If *θ*_*i*_ ∈ [0,1] is a numeric value, then the linguistic scale function *f* that conducts the mapping from *s*_*i*_ to *θ*_*i*_  (*i* = 0,1, 2,…, 2*t*) is defined as follows:

(4) $$\begin{array}{c} f:s_{i}⟶\theta_{i}\;\left(i=0,1,2,\ldots ,2t\right), \end{array}$$

where 0 ≤ *θ*_0_ < *θ*_1_ < ⋯<*θ*_2*t*_.

Clearly, the symbol *θ*_*i*_  (*i* = 0,1, 2,…, 2*t*) reflects the preference of the decision makers when they are using the linguistic term *s*_*i*_ ∈ *S*  (*i* = 0,1, 2,…, 2*t*). Therefore, the function/value in fact denotes the semantics of the linguistic terms.

 (1) Consider
  (5) $$\begin{array}{c} f_{1}\left(s_{i}\right)=\theta_{i}=\frac{i}{2t}\;\left(i=0,1,2,\ldots ,2t\right). \end{array}$$

The evaluation scale of the linguistic information given above is divided on average.

 (2) Consider
  (6) $$\begin{array}{c} f_{2}\left(s_{i}\right)=\theta_{i}=\{\begin{array}{cc} \frac{a^{t}-a^{t-i}}{2a^{t}-2} & \left(i=0,1,2,\ldots ,t\right),\\ \frac{a^{t}+a^{i-t}-2}{2a^{t}-2} & \left(i=t+1,t+2,\ldots ,2t\right). \end{array} \end{array}$$

With the extension from the middle of the given linguistic term set to both ends, the absolute deviation between adjacent linguistic subscripts also increases.

 (3) Consider
  (7) $$\begin{array}{c} f_{3}\left(s_{i}\right)=\theta_{i}=\{\begin{array}{cc} \frac{t^{\alpha }-{\left(t-i\right)}^{\alpha }}{2t^{\alpha }} & \left(i=0,1,2,\ldots ,t\right),\\ \frac{t^{\beta }+{\left(i-t\right)}^{\beta }}{2t^{\beta }} & \left(i=t+1,t+2,\ldots ,2t\right). \end{array} \end{array}$$

With the extension from the middle of the given linguistic term set to both ends, the absolute deviation between adjacent linguistic subscripts will decrease.

To preserve all the given information and facilitate the calculation, the above function can be expanded to $f^{*}:\overset{-}{S}\to R^{+}\;\;(R^{+}=\{r|r\geq 0,r\in R\})$, which satisfies f*(*s*_*i*_) = *θ*_*i*_, and is a strictly monotonically increasing and continuous function. Therefore, the mapping from $\overset{-}{S}$ to *R* ^+^ is one-to-one because of its monotonicity, and the inverse function of f* exists and is denoted by *f* ^∗−1^.

### 3.3. Operations of HFLNs

**Definition 7 (see [31]).**

Let *α* = 〈*s*_*θ*(*α*)_, *h*_*α*_〉 and *β* = 〈*s*_*θ*(*β*)_, *h*_*β*_〉 be two HFLNs. Some operations of *α* and *β* are defined as follows:

1. *α* ⊕ *β* = 〈*s*_*θ*(*α*)+*θ*(*β*)_, ∪_*r*_1_∈*h*_*α*_,*r*_2_∈*h*_*β*__{*r*_1_ + *r*_2_ − *r*_1_ *r*_2_}〉;
2. *λα* = 〈*s*_*λθ*(*α*)_, ∪_*r*∈*h*_*α*__{1 − (1 − *r*)^*λ*^}〉;
3. *α* ⊗ *β* = 〈*s*_*θ*(*α*)×*θ*(*β*)_, ∪_*r*_1_∈*h*_*α*_,*r*_2_∈*h*_*β*__{*r*_1_ *r*_2_}〉;
4. *α* ^*λ*^ = 〈*s*_*θ*(*α*)^*λ*^_, ∪_*r*∈*h*_*α*__{*r* ^*λ*^}〉.

The operations proposed in [31] have some obvious limitations. (a) All operations are carried out directly based on the subscripts of linguistic terms, which cannot reveal the critical differences of final results under various semantic situations. (b) The two parts of HFLNs are processed separately in the additive operation, that is, (1) of Definition 7, which may ignore the correlation of them. Take *α* = 〈*s*_2_, {0.3,0.4}〉; for example, 0.3 and 0.4 are the possible membership degrees that the object belongs to *s*_2_; that is, {0.3,0.4} is the explanatory part of *s*_2_ and should be closely related to *s*_2_ in the additive operation.

In order to overcome the existing limitations given above, new operations of HFLNs based on linguistic scale functions are defined as follows.

**Definition 8 .**

Let *α* = 〈*s*_*θ*(*α*)_, *h*_*α*_〉 and *β* = 〈*s*_*θ*(*β*)_, *h*_*β*_〉 be two HFLNs. Some operations of *α* and *β* are defined as follows:

(1) (8) $$\begin{array}{c} \text{neg}\left(\alpha \right)=\langle f^{*-1}\left(f^{*}\left(s_{2t}\right)-f^{*}\left(s_{\theta (\alpha )}\right)\right),\underset{r\in h_{\alpha }}{⋃}\left\{1-r\right\}\rangle , \end{array}$$
(2) (9) α⊕β=〈f∗−1(f∗(sθ(α))+f∗(sθ(β))),⋃r1∈hα,r2∈hβ{f∗(sθ(α))r1+f∗(sθ(β))r2f∗(sθ(α))+f∗(sθ(β))}〉,
(3) (10) $$\begin{array}{c} \lambda \alpha =\langle f^{*-1}\left(\lambda f^{*}\left(s_{\theta (\alpha )}\right)\right),h_{\alpha }\rangle ,\;\lambda \geq 0, \end{array}$$
(4) (11) $$\begin{array}{c} \alpha ⊗\beta =\langle f^{*-1}\left(f^{*}\left(s_{\theta (\alpha )}\right)f^{*}\left(s_{\theta (\beta )}\right)\right),\underset{r_{1}\in h_{\alpha },r_{2}\in h_{\beta }}{⋃}\left\{\begin{array}{cc} r_{1} & r_{2} \end{array}\right\}\rangle , \end{array}$$
(5) (12) $$\begin{array}{c} \alpha^{\lambda }=\langle f^{*-1}\left({\left(f^{*}\left(s_{\theta (\alpha )}\right)\right)}^{\lambda }\right),\underset{r\in h_{\alpha }}{⋃}\left\{r^{\lambda }\right\}\rangle ,\;\lambda \geq 0. \end{array}$$

According to Definition 6, it is known that f* is a mapping from the linguistic term *s*_*i*_ to the numeric value *θ*_*i*_ and *f* ^∗−1^ is a mapping from *θ*_*i*_ to *s*_*i*_. So, the first part of (1)–(5) is a linguistic term. In addition, it is obvious that the second part of (1)–(5) is an HFE. In summary, according to Definition 4, it is known that the results obtained by Definition 8 are also HFLNs.

The operations defined above are based on linguistic scale functions, which can get different results when a different linguistic scale function f* is utilized. Thus, decision makers can flexibly select the linguistic scale function f* depending on their preferences and the actual semantic situations. In addition, the new addition operation of HFLNs is more reasonable and reliable, because the final hesitant fuzzy membership has closely combined each element of the original HFLNs.

*α* ⊕ *β*, *λα*, *α* ⊗ *β*, and *α* ^*λ*^ necessarily appear in defining basic operations, but their results have no practical meaning. In the aggregation process, for example, using weighted operators, *α* ⊕ *β* is combined with *λα* and *α* ⊗ *β* is combined with *α* ^*λ*^; therefore, the calculation results are interpretable in practice.

**Example 9 .**

Let *S* = {*s*_0_, *s*_1_, *s*_2_, *s*_3_, *s*_4_, *s*_5_, *s*_6_} = {very poor, poor, slightly poor, fair, slightly good, good, very good}, *α* = 〈*s*_2_, {0.1,0.3}〉, *β* = 〈*s*_3_, {0.2}〉, and *λ* = 2.

If *f*_1_*(*s*_*i*_) = *i*/6  (0 ≤ *i* ≤ 6), then

1. neg(*α*) = 〈*s*_4_, {0.7,0.9}〉;
2. *α* ⊕ *β* = 〈*s*_5_, {0.16,0.24}〉;
3. 2*α* = 〈*s*_4_, {0.1,0.3}〉;
4. *α* ⊗ *β* = 〈*s*_1_, {0.02,0.06}〉;
5. *α* ^2^ = 〈*s*_0.667_, {0.01,0.09}〉.

If *a* = 1.4,

(13) $$\begin{array}{c} f_{2}^{*}\left(s_{i}\right)=\{\begin{array}{cc} \frac{a^{t}-a^{t-i}}{2a^{t}-2} & \left(0\leq i\leq t\right),\\ \frac{a^{t}+a^{i-t}-2}{2a^{t}-2} & \left(t<i\leq 2t\right), \end{array} \end{array}$$

then

1. neg(*α*) = 〈*s*_4_, {0.7,0.9}〉;
2. *α* ⊕ *β* = 〈*s*_5.532_, {0.156,0.244}〉;
3. 2*α* = 〈*s*_4.976_, {0.1,0.3}〉;
4. *α* ⊗ *β* = 〈*s*_0.835_, {0.02,0.06}〉;
5. *α* ^2^ = 〈*s*_0.622_, {0.01,0.09}〉.

If *α* = *β* = 0.8,

(14) $$\begin{array}{c} f_{3}^{*}\left(s_{i}\right)=\{\begin{array}{cc} \frac{t^{\alpha }-{\left(t-i\right)}^{\alpha }}{2t^{\alpha }} & \left(0\leq i\leq t\right),\\ \frac{t^{\beta }+{\left(i-t\right)}^{\beta }}{2t^{\beta }} & \left(t<i\leq 2t\right), \end{array} \end{array}$$

then

1. neg(*α*) = 〈*s*_4_, {0.7,0.9}〉;
2. *α* ⊕ *β* = 〈*s*_4.534_, {0.163,0.237}〉;
3. 2*α* = 〈*s*_3.326_, {0.1,0.3}〉;
4. *α* ⊗ *β* = 〈*s*_1.053_, {0.02,0.06}〉;
5. *α* ^2^ = 〈*s*_0.627_, {0.01,0.09}〉.

It can be easily proven that all the results given above are also HFLNs. In terms of the corresponding operations of HFLNs, the following theorem can also be proven easily.

**Theorem 10 .**

Let *α*_*i*_ = 〈*s*_*θ*(*α*_*i*_)_, *h*_*α*_*i*__〉  (*i* = 1,2, 3) be any three HFLNs; thus the following properties are true.

1. *α*_1_ ⊕ *α*_2_ = *α*_2_ ⊕ *α*_1_;
2. *α*_1_ ⊗ *α*_2_ = *α*_2_ ⊗ *α*_1_;
3. (*α*_1_ ⊕ *α*_2_) ⊕ *α*_3_ = *α*_1_ ⊕ (*α*_2_ ⊕ *α*_3_);
4. (*α*_1_ ⊗ *α*_2_) ⊗ *α*_3_ = *α*_1_ ⊗ (*α*_2_ ⊗ *α*_3_);
5. *λ*(*α*_1_ ⊕ *α*_2_) = *λα*_1_ ⊕ *λα*_2_, (*λ* ≥ 0);
6. (*α*_1_ ⊗ *α*_2_)^*λ*^ = *α*_1_^*λ*^ ⊗ *α*_2_^*λ*^, (*λ* ≥ 0).

**Proof**

According to Definition 8, it is known that Properties (1), (2), and (5) are obvious, so the proof of Property (3) is provided now.

 (3) Consider
  (15) (α1⊕α2)⊕α3 =〈f∗−1[f∗(sθ(α1))+f∗(sθ(α2))],⋃r1∈hα1,r2∈hα2{f∗(sθ(α1))r1+f∗(sθ(α2))r2f∗(sθ(α1))+f∗(sθ(α2))}〉  ⊕α3 =〈f∗−1{f∗[f∗−1(f∗(sθ(α1))+f∗(sθ(α2)))]+f∗(sθ(α3))},⋃r1∈hα1,r2∈hα2,r3∈hα3{(f∗{f∗−1[f∗(sθ(α1))+f∗(sθ(α2))]}×f∗(sθ(α1))r1+f∗(sθ(α2))r2f∗(sθ(α1))+f∗(sθ(α2))+f∗(sθ(α3))r3)×(f∗{f∗−1[f∗(sθ(α1))+f∗(sθ(α2))]}+f∗(sθ(α3)))−1}〉 =〈f∗−1{[f∗(sθ(α1))+f∗(sθ(α2))]+f∗(sθ(α3))},⋃r1∈hα1,r2∈hα2,r3∈hα3{([f∗(sθ(α1))+f∗(sθ(α2))]×f∗(sθ(α1))r1+f∗(sθ(α2))r2f∗(sθ(α1))+f∗(sθ(α2))+f∗(sθ(α3))r3)×([f∗(sθ(α1))+f∗(sθ(α2))]+f∗(sθ(α3)))−1}〉 =〈f∗−1[f∗(sθ(α1))+f∗(sθ(α2))+f∗(sθ(α3))],⋃r1∈hα1,r2∈hα2,r3∈hα3{(f∗(sθ(α1))r1+f∗(sθ(α2))r2+f∗(sθ(α3))r3)×(f∗(sθ(α1))+f∗(sθ(α2))+f∗(sθ(α3)))−1}〉,α1⊕(α2⊕α3) =α1⊕〈f∗−1[f∗(sθ(α2))+f∗(sθ(α3))],⋃r2∈hα2,r3∈hα3{f∗(sθ(α2))r2+f∗(sθ(α3))r3f∗(sθ(α2))+f∗(sθ(α3))}〉 =〈f∗−1{f∗(sθ(α1))+f∗[f∗−1(f∗(sθ(α2))+f∗(sθ(α3)))]},⋃r1∈hα1,r2∈hα2,r3∈hα3{(f∗(sθ(α1))r1+f∗{f∗−1[f∗(sθ(α2))+f∗(sθ(α3))]}×f∗(sθ(α2))r2+f∗(sθ(α3))r3f∗(sθ(α2))+f∗(sθ(α3)))×(f∗(sθ(α1))+f∗{f∗−1×[f∗(sθ(α2))+f∗(sθ(α3))]})−1}〉 =〈f∗−1{f∗(sθ(α1))+[f∗(sθ(α2))+f∗(sθ(α3))]},⋃r1∈hα1,r2∈hα2,r3∈hα3{(f∗(sθ(α1))r1+[f∗(sθ(α2))+f∗(sθ(α3))]×f∗(sθ(α2))r2+f∗(sθ(α3))r3f∗(sθ(α2))+f∗(sθ(α3)))×(f∗(sθ(α1))+[f∗(sθ(α2))+f∗(sθ(α3))])−1}〉 =〈f∗−1[f∗(sθ(α1))+f∗(sθ(α2))+f∗(sθ(α3))],⋃r1∈hα1,r2∈hα2,r3∈hα3{(f∗(sθ(α1))r1+f∗(sθ(α2))r2+f∗(sθ(α3))r3)×(f∗(sθ(α1))+f∗(sθ(α2))+f∗(sθ(α3)))−1}〉.

So, (*α*_1_ ⊕ *α*_2_) ⊕ *α*_3_ = *α*_1_ ⊕ (*α*_2_ ⊕ *α*_3_).

Similarity, Properties (4) and (6) can be easily proven.

### 3.4. Comparison Method for HFLNs

**Definition 11 .**

Let *α* = 〈*s*_*θ*(*α*)_, *h*_*α*_〉 be an HFLN. The score function *E*(*α*) of *α* can be represented as follows:

(16) $$\begin{array}{c} E\left(\alpha \right)=f^{*}\left(s_{\theta (\alpha )}\right)\times s\left(h_{\alpha }\right), \end{array}$$

where *s*(*h*_*α*_) is the score function of *h*_*α*_.

**Example 12 .**

Let *α* = 〈*s*_3_, {0.2,0.4,0.5,0.7}〉. If *t* = 3 and *f*_1_*(*s*_*i*_) = *i*/2*t*, by applying (16), then

(17) s(hα)=0.2+0.4+0.5+0.74=0.45,E(α)=36×0.45=0.225.

**Definition 13 .**

Let *α* = 〈*s*_*θ*(*α*)_, *h*_*α*_〉 = 〈*s*_*θ*(*α*)_, ∪_*r*∈*h*_*α*__{*r*}〉 be an HFLN. A variance function $\tilde{D}(h_{\alpha })$ of *h*_*α*_ can be denoted by $\tilde{D}(h_{\alpha })=\left(1/l(h_{\alpha })\right)\cdot \sum_{r\in h_{\alpha }}{\left[r-s(h_{\alpha })\right]}^{2}$. So, the accuracy function *D*(*α*) of *α* can be represented as follows:

(18) $$\begin{array}{c} D\left(\alpha \right)=f^{*}\left(s_{\theta (\alpha )}\right)\cdot \left[1-\tilde{D}\left(h_{\alpha }\right)\right], \end{array}$$

where *l*(*h*_*α*_) is the number of the values in *h*_*α*_.

**Example 14 .**

Let *α* = 〈*s*_3_, {0.3,0.6}〉. If *t* = 3 and *f*_1_*(*s*_*i*_) = *i*/2*t*, by applying (18), then

(19) D~(hα)=12×[(0.3−0.45)2+(0.6−0.45)2]=0.0225,D(α)=36×(1−0.0225)=0.489.

**Definition 15 .**

Let *α*_1_ = 〈*s*_*θ*(*α*_1_)_, *h*_*α*_1__〉 and *α*_2_ = 〈*s*_*θ*(*α*_2_)_, *h*_*α*_2__〉 be any two HFLNs.

1. If *E*(*α*_1_) > *E*(*α*_2_), then *α*_1_ > *α*_2_.
2. If *E*(*α*_1_) = *E*(*α*_2_), then
   if *D*(*α*_1_) > *D*(*α*_2_), then *α*_1_ > *α*_2_;
   if *D*(*α*_1_) = *D*(*α*_2_), then *α*_1_ = *α*_2_.

**Example 16 .**

Let *α* = 〈*s*_3_, {0.3,0.6}〉 and *β* = 〈*s*_6_, {0.15,0.3}〉. If *t* = 3 and *f*_1_*(*s*_*i*_) = *i*/2*t*, then *E*(*α*) = *E*(*β*) = 0.225, *D*(*α*) = 0.489, *D*(*β*) = 0.994, and thus *α* < *β*.

## 4. Hesitant Fuzzy Linguistic Prioritized Aggregation Operations and Their Applications in MCDM Problems

In this section, two prioritized aggregation operators for HFLNs are proposed based on the PA operator, and some desirable properties are also analyzed. Subsequently, these operators are extended to a generalized form. Finally, a method for solving MCDM problems with HFLNs, where the criteria are in different priority levels, is developed.

The PA operator was originally introduced by Yager [49] and is shown as follows.

**Definition 17 (see [49]).**

Let *G* = {*G*_1_, *G*_2_,…, *G*_*n*_} be a collection of criteria and ensure that there is a prioritization between the criteria expressed by the linear ordering *G*_1_≻*G*_2_≻*G*_3_≻⋯≻*G*_*n*_, which indicates that the criteria *G*_*j*_ has a higher priority than *G*_*k*_, if *j* < *k*. *G*_*j*_(*x*) is an evaluation value denoting the performance of the alternative *x* under the criteria *G*_*j*_ and satisfies *G*_*j*_(*x*)∈[0,1]. If

(20) $$\begin{array}{c} \text{PA}\left(G_{j}\left(x\right)\right)=\overset{n}{\underset{j=1}{\sum }}w_{j}G_{j}\left(x\right), \end{array}$$

where *w*_*j*_ = *T*_*j*_/∑_*i*=1_^*n*^ *T*_*i*_, *T*_1_ = 1 and *T*_*j*_ = ∏_*k*=1_^*j*−1^ *G*_*k*_(*x*)  (*j* = 2,…, *n*), then PA is called the PA operator.

PA operators have usually been used in situations where input arguments are exact values. Therefore, PA operators could be extended to accommodate situations where the input arguments are hesitant fuzzy linguistic information. Based on Definition 17, assume {*α*_1_, *α*_2_,…, *α*_*n*_} and {*β*_1_, *β*_2_,…, *β*_*n*_} are two sets of criteria values under criteria {*G*_1_, *G*_2_,…, *G*_*n*_}, where *G*_1_≻*G*_2_≻*G*_3_≻⋯≻*G*_*n*_. Now the PA operators under a hesitant fuzzy linguistic environment will be analyzed in the following subsections.

### 4.1. The Hesitant Fuzzy Linguistic Prioritized Weighted Average (HFLPWA) Operator

In this subsection, the prioritized weighted average operator under a hesitant fuzzy linguistic environment is investigated. The definition of the HFLPWA operator and its relevant theorems are given as follows.

**Definition 18 .**

Let *α*_*j*_  (*j* = 1,2,…, *n*) be a collection of HFLNs, and then the HFLPWA operator can be defined as follows:

(21) HFLPWA(α1,α2,…,αn) =T1∑i=1nTiα1⊕T2∑i=1nTiα2⊕⋯⊕Tn∑i=1nTiαn =  ⨁j=1n(Tjαj∑i=1nTi),

where *T*_*j*_ = ∏_*k*=1_^*j*−1^ *E*(*α*_*k*_)  (*j* = 2,…, *n*), *T*_1_ = 1, and *E*(*α*_*k*_) is the score function of *α*_*k*_.

Based on the operations of HFLNs described in Section 3, Theorem 19 can be deduced. The HFLPWA operator defined in Definition 18 is an abstract expression, whereas Theorem 19 gives the specific expression for it.

**Theorem 19 .**

Let *α*_*j*_ = *G*_*j*_(*x*) = 〈*s*_*θ*(*α*_*j*_)_, *h*_*α*_*j*__〉  (*j* = 1,2,…, *n*) be a collection of HFLNs. Then the aggregated value, obtained by using the HFLPWA operator, is also an HFLN, and

(22) HFLPWA(α1,α2,…,αn) =T1∑i=1nTiα1⊕T2∑i=1nTiα2⊕⋯⊕Tn∑i=1nTiαn =〈f∗−1(∑j=1n(Tj∑i=1nTif∗(sθ(αj)))),⋃r1∈hα1,…,rn∈hαn{∑j=1nf∗(sθ(αj))rjTj∑j=1nf∗(sθ(αj))Tj}〉,

where *T*_*j*_ = ∏_*k*=1_^*j*−1^ *E*(*α*_*k*_)  (*j* = 2,…, *n*), *T*_1_ = 1, and *E*(*α*_*k*_) is the score function of *α*_*k*_.

**Proof**

Clearly, according to Definition 8, the aggregated value is also an HFLN. In the following, (22) is proven by using a mathematical induction on *n*.

(1) For *n* = 2, since
  (23) T1∑i=1nTiα1=〈f∗−1(T1∑i=1nTif∗(sθ(α1))),hα1〉,T2∑i=1nTiα2=〈f∗−1(T2∑i=1nTif∗(sθ(α2))),hα2〉,
  we have
  (24) HFLPWA(α1,α2) =T1∑i=1nTiα1⊕T2∑i=1nTiα2 =〈f∗−1(T1∑i=1nTif∗(sθ(α1))+T2∑i=1nTif∗(sθ(α2))),⋃r1∈hα1,r2∈hα2{f∗(sθ(α1))r1T1+f∗(sθ(α2))r2T2f∗(sθ(α1))T1+f∗(sθ(α2))T2}〉.
(2) If (22) holds for *n* = *k*, then
  (25) HFLPWA(α1,α2,…,αk) =T1∑i=1nTiα1⊕T2∑i=1nTiα2⊕⋯⊕Tk∑i=1nTiαk =〈f∗−1(∑j=1k(Tj∑i=1nTif∗(sθ(αj)))),⋃r1∈hα1,…,rk∈hαk{∑j=1kf∗(sθ(αj))rjTj∑j=1kf∗(sθ(αj))Tj}〉.

When *n* = *k* + 1, by the operations described in Section 3, we have

(26) HFLPWA(α1,α2,…,αk+1) =〈f∗−1(∑j=1k(Tj∑i=1nTif∗(sθ(αj)))),⋃r1∈hα1,…,rk∈hαk{∑j=1kf∗(sθ(αj))rjTj∑j=1kf∗(sθ(αj))Tj}〉  ⊕〈f∗−1(Tk+1∑i=1nTif∗(sθ(αk+1))),hαk+1〉 =〈f∗−1(∑j=1k+1(Tj∑i=1nTif∗(sθ(αj)))),⋃r1∈hα1,…,rk+1∈hαk+1{∑j=1k+1f∗(sθ(αj))rjTj∑j=1k+1f∗(sθ(αj))Tj}〉;

that is, (22) holds for *n* = *k* + 1. Thus, (22) holds for all *n*. Now

(27) HFLPWA(α1,α2,…,αn) =T1∑i=1nTiα1⊕T2∑i=1nTiα2⊕⋯⊕Tn∑i=1nTiαn =〈f∗−1(∑j=1n(Tj∑i=1nTif∗(sθ(αj)))),⋃r1∈hα1,…,rn∈hαn{∑j=1nf∗(sθ(αj))rjTj∑j=1nf∗(sθ(αj))Tj}〉.

**Theorem 20 (boundedness).**

Let *α*_*j*_ = *G*_*j*_(*x*) = 〈*s*_*θ*(*α*_*j*_)_, *h*_*α*_*j*__〉  (*j* = 1,2,…, *n*) be a collection of HFLNs. If *α* ^−^ = 〈min⁡_*j*_{*s*_*θ*(*α*_*j*_)_}, *r* ^−^〉, *α* ^+^ = 〈max⁡_*j*_{*s*_*θ*(*α*_*j*_)_}, *r* ^+^〉, where *r* ^−^ = min⁡_*r*_*j*_∈∪_*h*_*α*_1__,*h*_*α*_2__,…,*h*_*α*_*n*____{*r*_*j*_} and *r* ^+^ = max⁡_*r*_*j*_∈∪_*h*_*α*_1__,*h*_*α*_2__,…,*h*_*α*_*n*____{*r*_*j*_}, then

(28) $$\begin{array}{c} E\left(\alpha^{-}\right)\leq E\left(HFLPWA\left(\alpha_{1},\alpha_{2},\ldots ,\alpha_{n}\right)\right)\leq E\left(\alpha^{+}\right). \end{array}$$

**Proof**

Let HFLPWA(*α*_1_, *α*_2_,…, *α*_*n*_) = *α* = 〈*s*_*θ*(*α*)_, *h*_*α*_〉, and then *E*(*α*) = f*(*s*_*θ*(*α*)_) · *s*(*h*_*α*_).

Since min⁡_*j*_{*s*_*θ*(*α*_*j*_)_} ≤ *s*_*θ*(*α*_*j*_)_ ≤ max⁡_*j*_{*s*_*θ*(*α*_*j*_)_} for all *j*, we have

(29) min⁡j{sθ(αj)} =f∗−1(∑k=1n(Tk∑i=1nTif∗(min⁡j{sθ(αj)}))) ≤f∗−1(∑k=1n(Tk∑i=1nTif∗(sθ(αk)))) =sθ(α) ≤f∗−1(∑k=1n(Tk∑i=1nTif∗(max⁡j{sθ(αj)}))) =max⁡j{sθ(αj)},

and then

(30) $$\begin{array}{c} \underset{j}{\min}\left\{s_{\theta (\alpha_{j})}\right\}\leq s_{\theta (\alpha )}\leq \underset{j}{\max}\left\{s_{\theta (\alpha_{j})}\right\}. \end{array}$$

Similarly, since

(31) s(r−)=r−=s(∑j=1nf∗(sθ(αj))Tj·r−∑j=1nf∗(sθ(αj))Tj)≤s(⋃r1∈hα1,…,rn∈hαn{∑j=1nf∗(sθ(αj))rjTj∑j=1nf∗(sθ(αj))Tj})=s(hα)≤s(∑j=1nf∗(sθ(αj))Tj·r+∑j=1nf∗(sθ(αj))Tj)=r+=s(r+),

therefore

(32) E(α−)=f∗(min⁡j{sθ(αj)})·s(r−)≤f∗(sθ(α))·s(hα)≤f∗(max⁡j{sθ(αj)})·s(r+)=E(α+).

So

(33) $$\begin{array}{c} E\left(\alpha^{-}\right)\leq E\left(\text{HFLPWA}\left(\alpha_{1},\alpha_{2},\ldots ,\alpha_{n}\right)\right)\leq E\left(\alpha^{+}\right). \end{array}$$

**Theorem 21 (commutativity).**

Let *α*_*j*_ = *G*_*j*_(*x*) = 〈*s*_*θ*(*α*_*j*_)_, *h*_*α*_*j*__〉  (*j* = 1,2,…, *n*) be a collection of HFLNs and $\left({\tilde{\alpha }}_{1},{\tilde{\alpha }}_{2},\ldots ,{\tilde{\alpha }}_{n}\right)$ be any permutation of (*α*_1_, *α*_2_,…, *α*_*n*_). Then

(34) $$\begin{array}{c} \text{HFLPWA}\left(\alpha_{1},\alpha_{2},\ldots ,\alpha_{n}\right)=\text{HFLPWA}\left({\tilde{\alpha }}_{1},{\tilde{\alpha }}_{2},\ldots ,{\tilde{\alpha }}_{n}\right). \end{array}$$

The weight of *α*_*j*_ is decided by the priority and value of *α*_*j*_ and will not be influenced by its position in the permutation. So, Theorem 21 can be easily proven, and the proof is therefore omitted.

It should be noted that the HFLPWA operator cannot satisfy idempotency. Take *α* = 〈*s*_2_, {0.2,0.4}〉, for example, and if *α*_1_ = *α*_2_ = *α*, then HFLPWA(*α*_1_, *α*_2_) = 〈*s*_2_, {0.2,0.218,0.382,0.4}〉 ≠ *α*. In addition, we do not consider the monotonicity of HFLPWA because the weights will be recalculated and vary if the values used in the HFLPWA operator change. It is difficult to consider the monotonic property when the parameters are irregularly variable.

### 4.2. The Hesitant Fuzzy Linguistic Prioritized Weighted Geometric (HFLPWG) Operator

In this subsection, the prioritized weighted geometric operator under a hesitant fuzzy linguistic environment is investigated. The definition of the HFLPWG operator and its relevant theorems are given as follows.

**Definition 22 .**

Let *α*_*j*_  (*j* = 1,2,…, *n*) be a collection of HFLNs, and then the HFLPWG operator can be defined as follows:

(35) HFLPWG(α1,α2,…,αn) =α1T1/∑i=1nTi⊗α2T2/∑i=1nTi⊗⋯⊗αnTn/∑i=1nTi =  ⨂j=1n(αjTj/∑i=1nTi),

where *T*_*j*_ = ∏_*k*=1_^*j*−1^ *E*(*α*_*k*_)  (*j* = 2,…, *n*), *T*_1_ = 1, and *E*(*α*_*k*_) is the score function of *α*_*k*_.

Similar to the HFLPWA operator, the HFLPWG operator satisfies the following properties.

**Theorem 23 .**

Let *α*_*j*_ = 〈*s*_*θ*(*α*_*j*_)_, *h*_*α*_*j*__〉  (*j* = 1,2,…, *n*) be a collection of HFLNs. Then the aggregated value, obtained by using the HFLPWG operator, is also an HFLN, and

(36) HFLPWG(α1,α2,…,αn) =〈f∗−1(∏j=1n(f∗(sθ(αj)))Tj/∑i=1nTi),⋃r1∈hα1,…,rn∈hαn{∏j=1n(rαj)Tj/∑i=1nTi}〉,

where *T*_*j*_ = ∏_*k*=1_^*j*−1^ *E*(*α*_*k*_)  (*j* = 2,…, *n*), *T*_1_ = 1, and *E*(*α*_*k*_) is the score function of *α*_*k*_.

**Proof**

Clearly, according to Definition 8, the aggregated value is also an HFLN. In the following, (36) is proven by using a mathematical induction on *n*.

(1) For *n* = 2, since
  (37) α1T1/∑i=1nTi=〈f∗−1((f∗(sθ(α1)))T1/∑i=1nTi),⋃r1∈hα1{r1T1/∑i=1nTi}〉,α2T2/∑i=1nTi=〈f∗−1((f∗(sθ(α2)))T2/∑i=1nTi),⋃r2∈hα2{r2T2/∑i=1nTi}〉

we have

(38) HFLPWG(α1,α2) =α1T1/∑i=1nTi⊗α2T2/∑i=1nTi =〈f∗−1((f∗(sθ(α1)))T1/∑i=1nTi·(f∗(sθ(α2)))T2/∑i=1nTi),⋃r1∈hα1,r2∈hα2{r1T1/∑i=1nTi·r2T2/∑i=1nTi}〉.

(2) If (36) holds for *n* = *k*, then
  (39) HFLPWG(α1,α2,…,αk) =α1T1/∑i=1nTi⊗α2T2/∑i=1nTi⊗⋯⊗αkTk/∑i=1nTi =〈f∗−1(∏j=1k(f∗(sθ(αj)))Tj/∑i=1nTi),⋃r1∈hα1,…,rk∈hαk{∏j=1k(rαj)Tj/∑i=1nTi}〉.

When *n* = *k* + 1, by the operations described in Section 3, we have

(40) HFLPWG(α1,α2,…,αk+1) =〈f∗−1(∏j=1k(f∗(sθ(αj)))Tj/∑i=1nTi),⋃r1∈hα1,…,rk∈hαk{∏j=1k(rαj)Tj/∑i=1nTi}〉  ⊗〈f∗−1((f∗(sθ(αk+1)))Tk+1/∑i=1nTi),⋃rk+1∈hαk+1{rk+1Tk+1/∑i=1nTi}〉 =〈f∗−1(∏j=1k+1(f∗(sθ(αj)))Tj/∑i=1nTi),⋃r1∈hα1,…,rk+1∈hαk+1{∏j=1k+1(rαj)Tj/∑i=1nTi}〉;

that is, (36) holds for *n* = *k* + 1. Thus, (36) holds for all *n*. Now

(41) HFLPWG(α1,α2,…,αn) =〈f∗−1(∏j=1n(f∗(sθ(αj)))Tj/∑i=1nTi),⋃r1∈hα1,…,rn∈hαn{∏j=1n(rαj)Tj/∑i=1nTi}〉.

**Theorem 24 (boundedness).**

Let *α*_*j*_ = *G*_*j*_(*x*) = 〈*s*_*θ*(*α*_*j*_)_, *h*_*α*_*j*__〉  (*j* = 1,2,…, *n*) be a collection of HFLNs. If *α* ^−^ = 〈min⁡_*j*_{*s*_*θ*(*α*_*j*_)_}, *r* ^−^〉, *α* ^+^ = 〈max⁡_*j*_{*s*_*θ*(*α*_*j*_)_}, *r* ^+^〉, where *r* ^−^ = min⁡_*r*_*j*_∈∪_*h*_*α*_1__,*h*_*α*_2__,…,*h*_*α*_*n*____{*r*_*j*_} and *r* ^+^ = max⁡_*r*_*j*_∈∪_*h*_*α*_1__,*h*_*α*_2__,…,*h*_*α*_*n*____{*r*_*j*_}, then

(42) $$\begin{array}{c} E\left(\alpha^{-}\right)\leq E\left(HFLPWG\left(\alpha_{1},\alpha_{2},\ldots ,\alpha_{n}\right)\right)\leq E\left(\alpha^{+}\right). \end{array}$$

**Proof**

Let HFLPWG(*α*_1_, *α*_2_,…, *α*_*n*_) = *α* = 〈*s*_*θ*(*α*)_, *h*_*α*_〉, and then *E*(*α*) = f*(*s*_*θ*(*α*)_) · *s*(*h*_*α*_).

Since min⁡_*j*_{*s*_*θ*(*α*_*j*_)_} ≤ *s*_*θ*(*α*_*j*_)_ ≤ max⁡_*j*_{*s*_*θ*(*α*_*j*_)_} for all *j*, we have

(43) min⁡j{sθ(αj)} =f∗−1(∏k=1n(f∗(min⁡j{sθ(αj)}))Tk/∑i=1nTi) ≤f∗−1(∏k=1n(f∗(sθ(αk)))Tk/∑i=1nTi) =sθ(α) ≤f∗−1(∏k=1n(f∗(max⁡j{sθ(αj)}))Tk/∑i=1nTi) =max⁡j{sθ(αj)},

and then

(44) $$\begin{array}{c} \underset{j}{\min}\left\{s_{\theta (\alpha_{j})}\right\}\leq s_{\theta (\alpha )}\leq \underset{j}{\max}\left\{s_{\theta (\alpha_{j})}\right\}. \end{array}$$

Similarly,

(45) s(r−)=r−=s(∏j=1n(r−)Tj/∑i=1nTi)≤s(⋃r1∈hα1,…,rn∈hαn{∏j=1n(rαj)Tj/∑i=1nTi})=s(hα)≤s(∏j=1n(r+)Tj/∑i=1nTi)=r+=s(r+),

and hence

(46) E(α−)=f∗(min⁡j{sθ(αj)})·s(r−)≤f∗(sθ(α))·s(hα)≤f∗(max⁡j{sθ(αj)})·s(r+)=E(α+).

So

(47) $$\begin{array}{c} E\left(\alpha^{-}\right)\leq E\left(\text{HFLPWG}\left(\alpha_{1},\alpha_{2},\ldots ,\alpha_{n}\right)\right)\leq E\left(\alpha^{+}\right). \end{array}$$

**Theorem 25 (commutativity).**

Let *α*_*j*_ = *G*_*j*_(*x*) = 〈*s*_*θ*(*α*_*j*_)_, *h*_*α*_*j*__〉  (*j* = 1,2,…, *n*) be a collection of HFLNs and $\left({\tilde{\alpha }}_{1},{\tilde{\alpha }}_{2},\ldots ,{\tilde{\alpha }}_{n}\right)$ is any permutation of (*α*_1_, *α*_2_,…, *α*_*n*_). Then

(48) $$\begin{array}{c} \text{HFLPWG}\left(\alpha_{1},\alpha_{2},\ldots ,\alpha_{n}\right)=\text{HFLPWG}\left({\tilde{\alpha }}_{1},{\tilde{\alpha }}_{2},\ldots ,{\tilde{\alpha }}_{n}\right). \end{array}$$

Similarly to Theorem 23, Theorem 25 can be easily proven, and the proof is therefore omitted.

Besides, the HFLPWG operator cannot satisfy idempotency. Take *α* = 〈*s*_2_, {0.2,0.4}〉, for example; if *α*_1_ = *α*_2_ = *α*, then HFLPWG(*α*_1_, *α*_2_) = 〈*s*_2_, {0.2,0.213,0.376,0.4}〉 ≠ *α*. Similarly, we do not consider the monotonicity of HFLPWG because of the variability of weights.

### 4.3. The Hesitant Fuzzy Linguistic Generalized Prioritized Weighted Aggregation (HFLGPWA) Operator

In general, the HFLPWA operator emphasizes the impact of the overall evaluation data and the compensation between different evaluation results, while the HFLPWG operator emphasizes the balance in the system and the coordination between different evaluation results. In this section, the generalized form of the HFLPWA and HFLPWG operators will be proposed, that is, the hesitant fuzzy linguistic generalized prioritized weighted aggregation (HFLGPWA) operator.

**Definition 26 .**

Let *α*_*j*_  (*j* = 1,2,…, *n*) be a collection of HFLNs, and then the HFLGPWA operator can be defined as follows:

(49) HFLGPWA(α1,α2,…,αn) =(T1∑i=1nTiα1λ⊕T2∑i=1nTiα2λ⊕⋯⊕Tn∑i=1nTiαnλ)1/λ =(⨁j=1n(Tj∑i=1nTiαjλ))1/λ,

where *λ* > 0, *T*_*j*_ = ∏_*k*=1_^*j*−1^ *E*(*α*_*k*_)  (*j* = 2,…, *n*), *T*_1_ = 1, and *E*(*α*_*k*_) is the score function of *α*_*k*_.

Obviously, the HFLPWA and HFLPWG operators are the special cases of the HFLGPWA operator.

(1) If *λ* → 0, then the HFLGPWA operator degenerates into the HFLPWG operator:
  (50) HFLGPWG(α1,α2,…,αn) =α1T1/∑i=1nTi⊗α2T2/∑i=1nTi⊗⋯⊗αnTn/∑i=1nTi.
(2) If *λ* = 1, then the HFLGPWA operator degenerates into the HFLPWA operator:
  (51) HFLGPWA(α1,α2,…,αn) =T1∑i=1nTiα1⊕T2∑i=1nTiα2⊕⋯⊕Tn∑i=1nTiαn.

### 4.4. An MCDM Method with HFLNs

In this subsection, the HFLGPWA operator will be applied to MCDM problems with hesitant fuzzy linguistic information.

For MCDM problems with hesitant fuzzy linguistic information, assume that there is a set of criteria {*C*_1_, *C*_2_,…, *C*_*n*_}, and the prioritization relationships that exist among them are *C*_1_≻*C*_2_≻⋯≻*C*_*n*_. Every criterion in *C*_*i*_ has a higher priority than every criterion in *C*_*j*_ if *i* < *j*. Under these criteria, there is a set of alternatives {*x*_1_, *x*_2_,…, *x*_*m*_} and the criteria values of the alternatives are expressed as HFLNs *C*_*j*_(*x*_*i*_)  (*i* = 1,…, *m*; *j* = 1,…, *n*). Suppose that *R* = (*C*_*j*_(*x*_*i*_))_*m*×*n*_ is the decision matrix. Subsequently, a ranking of alternatives is required.

In the following paragraphs, the HFLGPWA operator is applied to MCDM problems with hesitant fuzzy linguistic information.

This method involves the following steps.

**Step 1 (normalize the decision matrix).**

The common types of criteria in MCDM problems are maximizing criteria and minimizing criteria. For the minimizing criteria the negation operator in Definition 8 is utilized in order to normalize HFLNs.

For convenience, the normalized criteria values of *x*_*i*_  (*i* = 1,2,…, *m*) with respect to *C*_*j*_  (*j* = 1,2,…, *n*) are also denoted by *C*_*j*_(*x*_*i*_) = 〈*s*_*θ*(*C*_*ij*_)_, *h*_*C*_*ij*__〉.

**Step 2 (calculate the comprehensive evaluation values for each alternative).**

Obtain the comprehensive evaluation values *X*_*i*_  (*i* = 1,2,…, *m*) of *x*_*i*_ by applying the formula as follows:

(52) $$\begin{array}{c} X_{i}=\text{HFLGPWA}\left(C_{1}\left(x_{i}\right),C_{2}\left(x_{i}\right),\ldots ,C_{n}\left(x_{i}\right)\right). \end{array}$$

**Step 3 (calculate the score values and accuracy values of *X*_*i*_).**

Use Definition 11 to calculate the score values *E*(*X*_*i*_)  (*i* = 1,2,…, *m*) of the comprehensive values *X*_*i*_ of the alternatives *x*_*i*_  (*i* = 1,2,…, *m*), in order to rank all alternatives *x*_*i*_  (*i* = 1,2,…, *m*) and then select the best one(s). If the score values are *E*(*X*_*i*_) = *E*(*X*_*j*_)  (*i* ≠ *j*), it is necessary to calculate the accuracy values *D*(*X*_*i*_) and *D*(*X*_*j*_) and then rank the alternatives *x*_*i*_ and *x*_*j*_ in accordance with these accuracy values.

**Step 4 (rank all the alternatives and select the best one(s)).**

Use Definition 15 to rank all the alternatives and select the best one(s) in accordance with *E*(*X*_*i*_) and *D*(*X*_*i*_)  (*i* = 1,2,…, *m*).

## 5. Illustrative Example

### 5.1. Background

The following case is adapted from [42].

ABC Nonferrous Metals Co. Ltd. is a large state-owned company whose main business is producing and selling nonferrous metals. It is also the largest manufacturer of multispecies nonferrous metals in China, with the exception of aluminum. To expand its main business, the company is always engaged in overseas investment, and a department which consists of executive managers and several experts in the field has been established specifically to make decisions on global mineral investment.

Recently, the overseas investment department decided to select a pool of alternatives from several foreign countries based on preliminary surveys. After thorough investigation, five countries (alternatives) are taken into consideration, that is, {*x*_1_, *x*_2_, *x*_3_, *x*_4_, *x*_5_}. There are many factors that affect the investment environment and four factors are considered based on the experience of the department personnel, including *C*_1_: resources (such as the suitability of the minerals and their exploration); *C*_2_: politics and policy (such as corruption and political risks); *C*_3_: economy (such as development vitality and the stability); *C*_4_: infrastructure (such as railway and highway facilities).

The decision makers, including the experts and executive managers, have gathered to determine the decision information. The linguistic term set *S* = {*s*_0_, *s*_1_, *s*_2_, *s*_3_, *s*_4_, *s*_5_, *s*_6_} = {very poor, poor, slightly poor, fair, slightly good, good, very good} is used. The evaluation information is given in the form of HFLNs, where *C*_*j*_(*x*_*i*_) is the evaluation value of the alternative *x*_*i*_ on the criterion *C*_*j*_. In *C*_*j*_(*x*_*i*_) there is a consensus on the chosen linguistic term and each decision maker can use a value to express his/her opinion; in other words, the value denotes to what degree *x*_*i*_ matches this given linguistic term under *C*_*j*_. Each decision maker gave his/her own evaluations (membership degrees) based on the surveys of the five countries as well as his/her knowledge and experience. Then all the possible membership degrees under each given linguistic term are gathered together. The same membership degrees for a given linguistic term will appear only once in an HFLN. Consequently, following a heated discussion, they came to a consensus on the final evaluations which are expressed by HFLNs as shown in Table 1.

### 5.2. An Illustration of the Proposed Method

Assume that the prioritization relationship for the criteria is *C*_1_≻*C*_2_≻*C*_3_≻*C*_4_.

To get the optimal alternative(s), let *f*_1_*(*s*_*i*_) = *i*/2*t* and adopt the following steps.

**Step 1 (normalize the decision matrix).**

Considering that all the criteria are of maximizing type, the performance values of the alternatives *x*_*i*_  (*i* = 1,2,…, 5) do not need to be normalized.

**Step 2 (calculate the comprehensive evaluation values for each alternative).**

Use Definition 26 and then the comprehensive evaluation values of the alternatives are obtained and are shown in Table 2.

**Step 3 (calculate the score values of *X*_*i*_).**

The score values *E*(*X*_*i*_)  (*i* = 1,2,…, 5) of the comprehensive evaluation values *X*_*i*_ of the alternatives *x*_*i*_  (*i* = 1,2,…, 5) can be calculated and are shown in Table 3.

It has been ascertained that, with the increase of *λ*, the score values *E*(*X*_*i*_) would increase slightly, where *i* = 1,2, 3,4, 5.

**Step 4 (rank all the alternatives and select the best one(s)).**

In accordance with the score values *E*(*X*_*i*_)  (*i* = 1,2,…, 5), the rankings of the alternatives are shown in Table 4 (note that “≻” means “preferred to”).

From Table 4 we can see that the rankings obtained by the HFLPWG and HFLPWA operators are the same. In addition, the alternative *x*_5_ is the best choice. This result can reveal the stability of the proposed method.

In order to illustrate the influence of the linguistic scale function f* and parameter *λ* on the decision-making process in this example, different f* and *λ* are used in Steps 2 and 3 to rank the alternatives. The ranking results are shown in Tables 5, 6, and 7.

We can conclude that the rankings of the alternatives may be a little different when a different linguistic scale function f* or parameter *λ* is utilized. If *λ* ≤ 5, the best alternative is *x*_5_; if *λ* ≥ 8, the best one is *x*_3_. Moreover, the worst alternative is always *x*_4_ except for one situation.

### 5.3. Comparison Analysis and Discussion

To further illustrate the advantages of the proposed MCDM approach under a hesitant fuzzy linguistic environment, the method in [31] is used to solve the same illustrative example given above.

Lin et al. [31] utilized the hesitant fuzzy linguistic weighted average (HFLWA) operator in order to obtain the comprehensive overall of alternatives. Their aggregation operator is now used with the scores of all alternatives being calculated as follows:

(53) $$\begin{array}{c} S\left(x_{1}\right)=1.720,\;\;S\left(x_{2}\right)=1.083,\;\;S\left(x_{3}\right)=1.632,\\ S\left(x_{4}\right)=1.022,\;\;S\left(x_{5}\right)=2.974. \end{array}$$

Since *S*(*x*_5_) > *S*(*x*_1_) > *S*(*x*_3_) > *S*(*x*_2_) > *S*(*x*_4_), the ranking is *x*_5_≻*x*_1_≻*x*_3_≻*x*_2_≻*x*_4_, and the most desirable car is *x*_5_.

Obviously, the rankings obtained by the proposed method in this paper may be a little different from that obtained by the method in [31]. The only difference is the order of *x*_1_ and *x*_3_, and this may be caused by the different operations and comparison method for HFLNs. The operations and comparison method for HFLNs in [31] consider only one semantic situation, while different linguistic scale functions f* used in this paper are applicable and effective under different semantic environment. In addition, the HFLWA operator in [31] emphasized the impact of the overall criterion values and the compensation between different criterion values, while the proposed hesitant fuzzy linguistic PA operators do not.

According to the above comparison analyses, the proposed method for MCDM problems with HFLNs has the following advantages.

First, HFLNs used in this paper can express the evaluation information more flexibly. They can depict fuzzy linguistic information more accurately and retain the completeness of the original data or the inherent thoughts of decision makers, which is the prerequisite of guaranteeing accuracy of final outcomes.

Second, the operations of HFLNs in this paper are defined based on linguistic scale functions, which can achieve different results when a different linguistic scale function f* is used. Thus, decision makers can flexibly select the linguistic scale function f* depending on their preferences and the actual semantic situations.

Third, the proposed hesitant fuzzy linguistic prioritized aggregation operators can deal with MCDM problems under the hesitant fuzzy linguistic environment in which the criteria are in different priority levels. What is more, the criteria weights, which are calculated by the prioritized aggregation operator according to the criteria priority levels, are more objective and reasonable than a set of known criteria weights.

## 6. Conclusions

To address situations where decision-making problems use qualitative variables rather than numerical ones and to reflect the uncertainty, hesitancy, and inconsistency of decision makers, HFLSs have been introduced and used in this paper. Considering the limitations in the existing literature, new operations of HFLNs were introduced. Then, on the basis of the PA operator, two prioritized aggregation operators for HFLNs were proposed and extended to a generalized form. Furthermore, an MCDM method based on the generalized prioritized aggregation operator under a hesitant fuzzy linguistic environment was developed. Finally, an illustrative example demonstrated the application of the proposed method and comparison analysis was made with the representative method. The results indicated that the method proposed in this paper is feasible and effective in solving MCDM problems with HFLSs.

It is well known that the PA operator has many advantages over other operators as it does not need to provide weight vectors and, when using this approach, it is only necessary to know the priority among criteria. The foremost characteristic of these proposed operators is that they take into account the priority among criteria. Although traditional prioritized aggregation operators are generally suitable for aggregating information which is in the form of numerical values or simple fuzzy values, they are unable to deal with hesitant fuzzy linguistic information. The proposed HFLGPWA operator can accommodate situations where the input arguments consist of hesitant fuzzy linguistic information. In addition, the results may change using different linguistic scale functions, and the parameter *λ* may also influence the results. Decision makers can select the most appropriate linguistic scale function f* according to their interests and actual semantic situations. In a word, the main advantages of the proposed method are not only that the proposed operators accommodate a hesitant fuzzy linguistic environment, but also its consideration of the priority among criteria, which is more feasible and practical. In the future research, the linguistic scale function can be applied in other linguistic sets, such as ILSs, LHFSs, and HFLSs. Moreover, we believe that the study of information measures and outranking relations for HFLSs does make great sense.

## Figures and Tables

**Table 1:** The hesitant fuzzy linguistic decision matrix.

	*C*_1_	*C*_2_	*C*_3_	*C*_4_
*x*_1_	〈*s*_4_, {0.4}〉	〈*s*_5_, {0.25,0.45}〉	〈*s*_4_, {0.55,0.75}〉	〈*s*_3_, {0.45}〉
*x*_2_	〈*s*_2_, {0.25,0.65}〉	〈*s*_4_, {0.35,0.55}〉	〈*s*_3_, {0.7}〉	〈*s*_4_, {0.35,0.55}〉
*x*_3_	〈*s*_3_, {0.35,0.6}〉	〈*s*_4_, {0.4}〉	〈*s*_6_, {0.75,0.85}〉	〈*s*_2_, {0.65}〉
*x*_4_	〈*s*_2_, {0.35,0.45,0.55}〉	〈*s*_3_, {0.6}〉	〈*s*_2_, {0.4,0.7}〉	〈*s*_2_, {0.85}〉
*x*_5_	〈*s*_5_, {0.45,0.8}〉	〈*s*_5_, {0.5}〉	〈*s*_3_, {0.6,0.85}〉	〈*s*_4_, {0.75}〉

**Table 2:** The aggregation results by utilizing the HFLGPWA operator.

	*λ* → 0 (HFLPWG)	*λ* = 1 (HFLPWA)
*X*_1_	〈*s*_4.15_, {0.373,0.380,0.418,0.425}〉	〈*s*_4.17_, {0.374,0.385,0.421,0.431}〉
*X*_2_	〈*s*_2.23_, {0.272,0.274,0.288,0.289,0.599,0.602,0.633,0.637}〉	〈*s*_2.31_, {0.295,0.300,0.338,0.343,0.581,0.586,0.624,0.629}〉
*X*_3_	〈*s*_3.21_, {0.380,0.382,0.566,0.570}〉	〈*s*_3.28_, {0.402,0.410,0.571,0.580}〉
*X*_4_	〈*s*_2.1_, {0.378,0.387,0.466,0.476,0.551,0.563}〉	〈*s*_2.12_, {0.399,0.410,0.477,0.488,0.555,0.566}〉
*X*_5_	〈*s*_4.66_, {0.491,0.512,0.674,0.702}〉	〈*s*_4.72_, {0.488,0.507,0.692,0.711}〉

**Table 3:** The score values for the alternatives.

	*λ* → 0 (HFLPWG)	*λ* = 1 (HFLPWA)
*E*(*X*_1_)	0.276	0.280
*E*(*X*_2_)	0.167	0.178
*E*(*X*_3_)	0.254	0.268
*E*(*X*_4_)	0.165	0.171
*E*(*X*_5_)	0.461	0.471

**Table 4:** Rankings of the alternatives.

	Ranking
*λ* → 0 (HFLPWG)	*x*_5_≻*x*_1_≻*x*_3_≻*x*_2_≻*x*_4_
*λ* = 1 (HFLPWA)	*x*_5_≻*x*_1_≻*x*_3_≻*x*_2_≻*x*_4_

**Table 5:** Rankings of the alternatives with different *λ* by using *f*_1_*.

	*λ* → 0; *λ* = 1	*λ* = 2; *λ* = 3; *λ* = 4; *λ* = 5; *λ* = 6	*λ* = 7; *λ* = 8; *λ* = 9; *λ* = 10
*f*_1_*	*x*_5_≻*x*_1_≻*x*_3_≻*x*_2_≻*x*_4_	*x*_5_≻*x*_3_≻*x*_1_≻*x*_2_≻*x*_4_	*x*_3_≻*x*_5_≻*x*_1_≻*x*_2_≻*x*_4_

**Table 6:** Rankings of the alternatives with different *λ* by using *f*_2_*.

	*λ* → 0	*λ* = 1; *λ* = 2; *λ* = 3; *λ* = 4; *λ* = 5	*λ* = 6; *λ* = 7; *λ* = 8; *λ* = 9; *λ* = 10
*f*_2_*	*x*_5_≻*x*_1_≻*x*_3_≻*x*_4_≻*x*_2_	*x*_5_≻*x*_3_≻*x*_1_≻*x*_2_≻*x*_4_	*x*_3_≻*x*_5_≻*x*_1_≻*x*_2_≻*x*_4_

**Table 7:** Rankings of the alternatives with different *λ* by using *f*_3_*.

	*λ* → 0; *λ* = 1; *λ* = 2	*λ* = 3; *λ* = 4; *λ* = 5; *λ* = 6; *λ* = 7	*λ* = 8; *λ* = 9; *λ* = 10
*f*_3_*	*x*_5_≻*x*_1_≻*x*_3_≻*x*_2_≻*x*_4_	*x*_5_≻*x*_3_≻*x*_1_≻*x*_2_≻*x*_4_	*x*_3_≻*x*_5_≻*x*_1_≻*x*_2_≻*x*_4_
